# NPAS2 dampens chemo-sensitivity of lung adenocarcinoma cells by enhancing DNA damage repair

**DOI:** 10.1038/s41419-023-06256-3

**Published:** 2024-01-30

**Authors:** Youyu Zhang, Yuqiao Chen, Wentao Huang, Yuan Zhou, Ya Wang, Kai Fu, Wei Zhuang

**Affiliations:** 1 Institute of Molecular Precision Medicine and Hunan Key Laboratory of Molecular Precision Medicine, Department of General Surgery, Xiangya Hospital, Central South University, 410008 Changsha, Hunan China; 2 Department of Thoracic Surgery, Xiangya Hospital, Central South University, 410008 Changsha, Hunan China; 3 Department of Cardiothoracic Vascular Surgery, Zhuzhou Central Hospital, 412001 Zhuzhou, Hunan China; 4 MOE Key Lab of Rare Pediatric Diseases & Hunan Key Laboratory of Medical Genetics of the School of Life Sciences, Central South University, 410031 Changsha, Hunan China; 5 National Clinical Research Center for Geriatric Disorders, 410008 Changsha, Hunan China

**Keywords:** Apoptosis, Non-small-cell lung cancer

## Abstract

Chemotherapeutic agents, including cisplatin, have remained a cornerstone of lung adenocarcinoma (LUAD) treatment and continue to play an essential role in clinical practice, despite remarkable progress in therapeutic strategies. Hence, a thorough comprehension of the molecular mechanisms underlying chemotherapeutic agent resistance is paramount. Our investigation centered on the potential involvement of the NPAS2 gene in LUAD, which is highly expressed in tumors and its high expression has been associated with unfavorable overall survival rates in patients. Intriguingly, we observed that the depletion of NPAS2 in LUAD cells resulted in increased susceptibility to cisplatin treatment. Furthermore, mRNA sequencing analysis revealed that NPAS2 deficiency downregulated genes crucial to DNA repair. Additionally, NPAS2 depletion significantly impairs γH2AX accumulation, a pivotal component of the DNA damage response. Further investigation demonstrates that NPAS2 plays a crucial role in DNA double-strand breakage repair via homology-directed repair (HDR). Our inquiry into the molecular mechanisms underlying NPAS2 regulation of DDR revealed that it may enhance the stability of H2AX mRNA by binding to its mRNA, thereby upregulating the DNA damage repair pathway. In-vivo experiments further confirmed the crucial role of NPAS2 in modulating the effect of cisplatin in LUAD. Taken together, our findings suggest that NPAS2 binds to and enhances the stability of H2AX mRNA, thereby decreasing the sensitivity of tumor cells to chemotherapy by augmenting DNA damage repair.

## Introduction

Although major advances in the treatment of lung adenocarcinoma (LUAD) have been made with the development of targeted agents such as epidermal growth factor receptor tyrosine kinase inhibitor (EGFR TKI) and immunotherapy, LUAD remains one of the most lethal malignancies [1]. Platinum-based doublet chemotherapy is still the current standard of care for advanced non-small-cell lung cancer (NSCLC) patients who do not have an epidermal growth factor receptor mutation or anaplastic lymphoma kinase (ALK) rearrangement [2–4]. Furthermore, the vast majority of patients develop drug resistance after initial treatment, and platinum-based doublet chemotherapy remains the preferred treatment strategy for these resistant patients. It is necessary to gain a better understanding of the molecular mechanisms of chemotherapy resistance and identify predictive markers.

Neuronal PAS domain protein 2 (NPAS2) is a pivotal circadian rhythm gene that is involved in the regulation of biological rhythms and plays a significant role in the regulation of cellular growth and differentiation [5, 6]. More recently, research has demonstrated that NPAS2 is not only implicated in the regulation of biological rhythms but also closely linked to tumorigenesis. NPAS2 can impact the occurrence and development of tumors through multiple pathways. For example, the binding of NPAS2 to the promoter of CDC25A leads to its trans-activation, thereby promoting the growth of hepatocellular carcinoma cells [7]. Additionally, the binding of NPAS2 to the promoter of HIF-1α mediates the reprogramming process of glucose metabolism in liver cancer cells [8].

Cisplatin, the fundamental drug used in the treatment of LUAD, initially causes interstrand crosslinks in the cells. If not properly repaired, interstrand crosslinks can result in persistent double-strand breaks (DSBs), the most dangerous of all DNA lesions, and finally lead to apoptosis [9, 10]. Homology-directed repair (HDR) is one of the primary pathways for cells to repair DSBs [11]. HDR employs the sister chromosome sequence as a template to repair the DSBs more accurately with the assistance of multiple DNA damage repair censors, such as H2AX, MRN complex (Mre11–Rad50–Nbs1) and Ataxia-telangiectasia mutated (ATM) kinase. Among these, H2AX plays a crucial role in the initiation and progression of HDR by promoting the recruitment and activation of DNA repair factors at sites of DNA damage, amplifying the DNA damage signal for easier detection and repair [12]. In a range of tumors, such as lung cancer, ovarian cancer, and nasopharyngeal carcinoma, abnormal activation of HDR can lead to cisplatin resistance in tumor cells [13–15].

The present investigation has yielded novel insights into the biological functions of the NPAS2 gene in LUAD. Specifically, we observed a significant upregulation of NPAS2 expression in this disease, which was associated with a poor prognosis. To gain a deeper understanding of the role of NPAS2 in this context, we performed RNA sequencing on PC-9 cells in which NPAS2 had been knocked down. Our findings showed that NPAS2 knockdown specifically inhibited DNA damage repair-related pathways, especially the HDR pathway. In addition, we observed that silencing NPAS2 led to enhanced sensitivity of lung adenocarcinoma cells to cisplatin and inhibition of DNA damage repair. Further investigation into the molecular mechanisms underlying NPAS2’s regulation of DNA damage response (DDR) revealed that it may bind and stabilize mRNA of H2AX, thereby upregulating the DNA damage repair pathway.

## Results

### NPAS2 is upregulated in LUAD tumor tissues and high expression of NPAS2 indicates a shorter overall survival

**Fig. 1:**
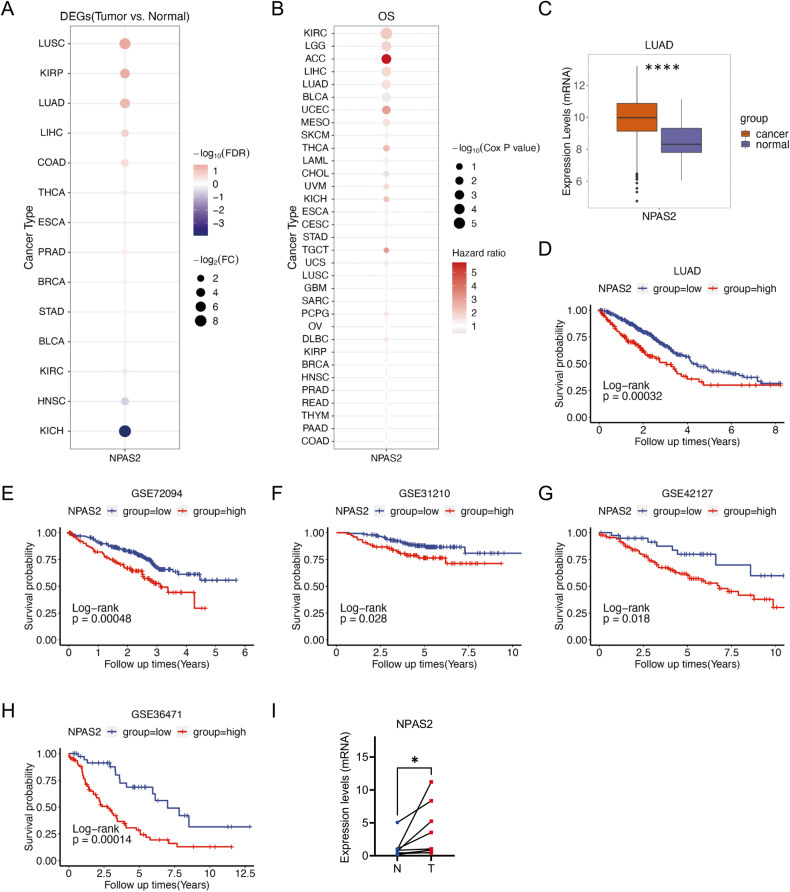
NPAS2 is increased in LUAD tumor tissues, and high expression of NPAS2 is associated with reduced overall survival. **A** The dot plot (left) shows the expression of NPAS2 in tumor and normal tissue in the TCGA pan-cancer dataset. The color of the dot represents the Log_10_(false discovery rate (FDR)), and the size of the dots represents the −Log_2_(Fold Change (FC)) of the expression of NPAS2 between tumor tissue and normal tissue. **B** The dot plot (Right) shows the results of univariate Cox regression analysis, which was used to assess the relationship between NPAS2 expression and prognosis in the TCGA pan-cancer dataset. The color of the dot represents the Hazard ratio of the expression of NPAS2. The size of the dot represents the −log_10_(Cox *P* value). **C** The boxplot showed the expression of NPAS2 in the tumor and normal tissue in TCGA LUAD. **D** Kaplan–Meier survival curves showed the overall survival between groups with low NPAS2 expression and high NPAS2 expression in the TCGA LUAD dataset. (Blue line indicates the group with low NPAS2 expression. Red line indicates the group with high NPAS2 expression.) **E**–**H** Kaplan–Meier survival curves showed the overall survival between groups with low NPAS2 expression and high NPAS2 expression in patients with LUAD in GEO datasets including GSE72094, GSE31210, GSE42127, and GSE36471. **I** The comparison between the expression levels of NPAS2 in tumor tissues and paired normal tissue from LUAD patients in our facility. ns non-significant difference; *p < 0.05; *p* > 0.05; ****p < 0.0001.

The expression of NPAS2 mRNA in tumor and normal tissues was first compared in TCGA pan-cancer datasets. The expression of NPAS2 was significantly lower in tumor samples when compared to normal samples in LUAD, LUSC, KIRP, and LIHC (Fig. 1A). The univariate Cox of overall survival (OS) was performed to evaluate if the differential expression of NPAS2 was related to the prognosis of patients with different cancer types. As shown in Fig. 1B, the high expression of NPAS2 indicates a shorter OS of patients with LUAD (HR = 1.61, *p* < 0.001), KIRC (hazard ratio (HR) = 2.08, *p* < 0.001), LGG (HR = 1.90, *p* < 0.001), ACC (HR = 5.73, *p* = 0.001) and LIHC (HR = 1.85, *p* < 0.001). Taken together, these results suggest that NPAS2 is highly expressed in LUAD, and its high expression predicts a poor prognosis. Therefore, further investigation was conducted to explore the role of NPAS2 in LUAD.

A boxplot was utilized to visually depict the marked upregulation of NPAS2 in tumor tissues of lung adenocarcinoma in comparison to the adjacent paracancerous tissues from the TCGA LUAD dataset (Fig. 1C). Additionally, Kaplan–Meier survival analysis further revealed that elevated NPAS2 expression was strongly correlated with poorer prognosis in LUAD patients in TCGA (Fig. 1D). Further examination of the association between NPAS2 expression and overall survival was conducted in the GEO datasets, where the results of Kaplan–Meier survival curves demonstrated a statistically significant association between high NPAS2 expression and shorter overall survival in patients with LUAD, as evidenced in GSE72094, GSE31210, GSE42127, and GSE36471 (Fig. 1E–H). Furthermore, RT-qPCR analysis performed on tumor and normal tissues obtained from our clinical facility confirmed a significant elevation of NPAS2 expression levels in the tumors from LUAD patients (Fig. 1I).

### mRNA sequencing indicates an attenuated DNA repair in NPAS2 deficiency LUAD cells

**Fig. 2:**
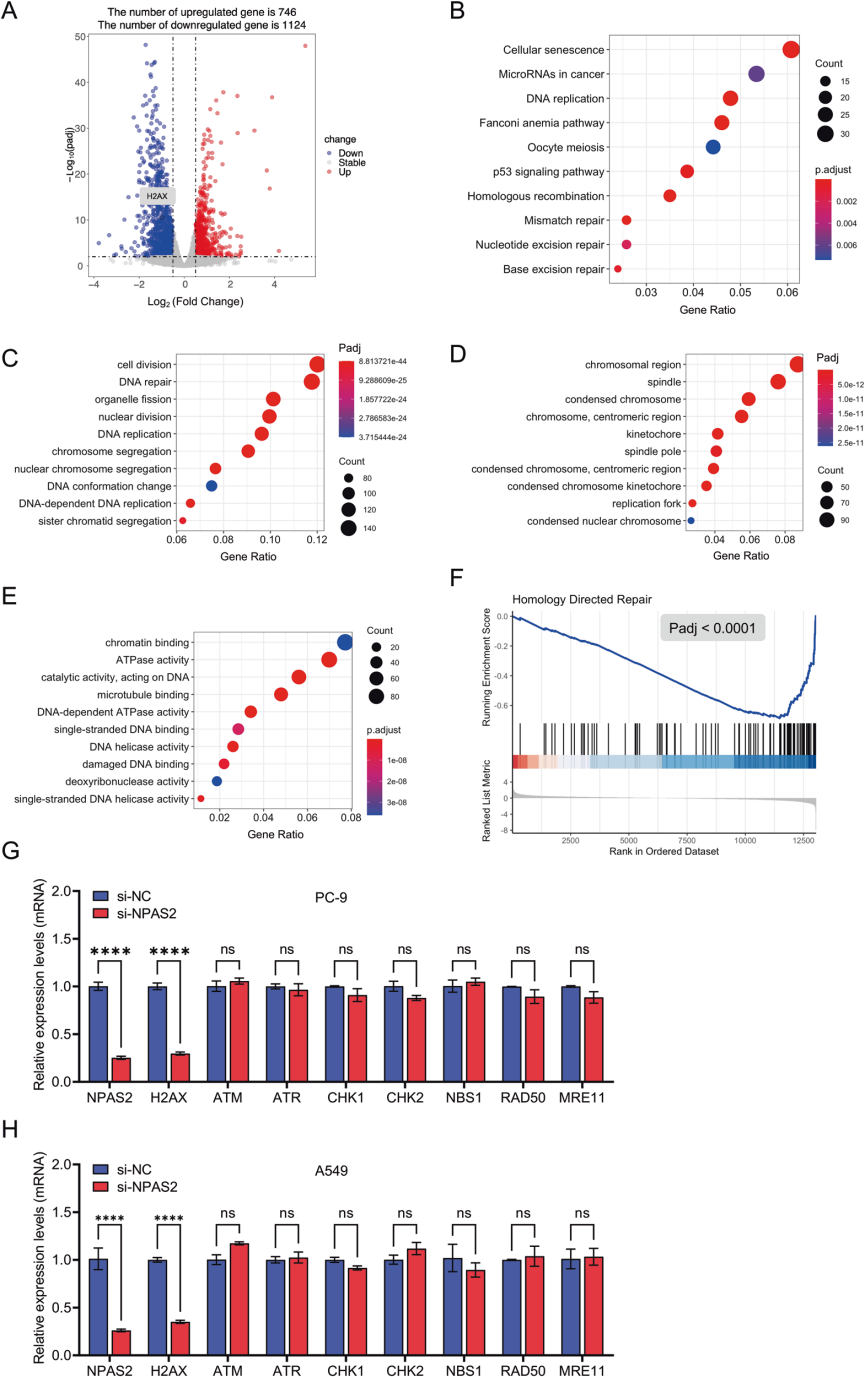
Global remodeling of gene expression in PC cells with knockdown of NPAS2. **A** The volcano plot shows the differential expression of genes between PC-9 cells transfected with si-NPAS2 and si-NC. *X*-axis represents the Fold Change in gene expression, *Y*-axis represents the Benjamini–Hochberg adjusted *P*-value. **B**–**E** KEGG and GO analysis of the downregulated genes after the silencing of NPAS2. **F** The GSEA analysis of the downregulated genes after the depletion of NPAS2 showed that the homologous recombination was highly enriched. **G** and **H** PC-9 (**G**) and A549 (**H**) cells were transfected for 72 h with si-NC or si-NPAS2. RT-qPCR was used to determine the relative mRNA levels of the indicated genes. ns non-significant difference; *p* > 0.05; ****p < 0.0001.

A comprehensive analysis of differential gene expression was conducted between PC-9 samples transfected with nonspecific control (si-NC) or NPAS2-specific (si-NPAS2) small interference RNAs, revealing 1870 differentially expressed genes (DEGs), including 746 upregulated and 1124 downregulated genes, as depicted in the volcano plots in Fig. 2A. Further exploration through KEGG pathway analysis highlighted the significant downregulation of genes involved in the vital processes of cell cycle, DNA replication, and homologous recombination (Fig. 2B). The analysis of GO annotations showed that the downregulated genes were enriched in DNA helicase activity and damaged DNA binding (ontology: biological process), chromosomal region and spindle (ontology: cellular component), and cell division and DNA repair (ontology: molecular function) (Fig. 2C–E). Additionally, the gene set enrichment analysis (GSEA) suggested that downregulated genes were highly enriched in HDR. All these results indicate a potential role for NPAS2 in DNA damage response and repair especially the HDR (Fig. 2F).

HDR, a process of repairing DNA double-strand break by homologous recombination using a DNA template, has been identified as one of the primary mechanisms for repairing DSBs [16]. Numerous crucial DNA damage repair-related proteins are required for the activation of the HDR signaling pathway (including MRE11, RAD50, NBS1, ATM, Chk2, H2AX, and other proteins). MRE11, RAD50, and NBS1 constitute the MRN complex, which recruits ATM to the site of DNA damage via direct interactions, and then activates ATM kinase [17, 18]. Activated ATM phosphorylates downstream substrates involved in DNA repair, cell cycle checkpoint control, and apoptosis. In the context of HDR repair, ATM phosphorylates and activates key HDR-related proteins, such as BRCA1 and RAD51, which are recruited to the DSB sites to promote strand invasion and homology search [19, 20]. ATR kinase is activated by ssDNA generated during DSBs repair and coordinates the activation of cell cycle checkpoints and DNA repair pathways, including HDR. Chk1 and Chk2 kinases are downstream effectors of the ATR and ATM kinases, respectively, and play important roles in regulating the cell cycle and promoting DNA repair. Chk1 is activated by ATR in response to DNA damage and regulates the G2/M checkpoint to prevent cells from entering mitosis until DNA damage is repaired. Chk2 is activated by ATM in response to DNA damage and regulates the G1/S checkpoint to prevent cells from replicating damaged DNA. H2AX is a histone variant that becomes phosphorylated on Ser139 (γH2AX) in response to DSBs, creating a platform for the recruitment of repair factors to the damaged site [21].

To corroborate those findings in mRNA sequencing, we investigated the influence of NPAS2 on the expression of key regulators in HDR, including MRE11, NBS1, RAD50, ATM, ATR, Chk1, Chk2, and H2AX, through RT-qPCR in PC-9 and A549 cells. The results indicated that NPAS2 knockdown significantly reduced the mRNA level of H2AX, while having no effect on the mRNA levels of MRE11, NBS1, RAD50, ATM, ATR, Chk1, and Chk2 (Fig. 2G, H).

### NPAS2 depletion impairs HDR by inhibiting the DNA damage repair signaling cascade

**Fig. 3:**
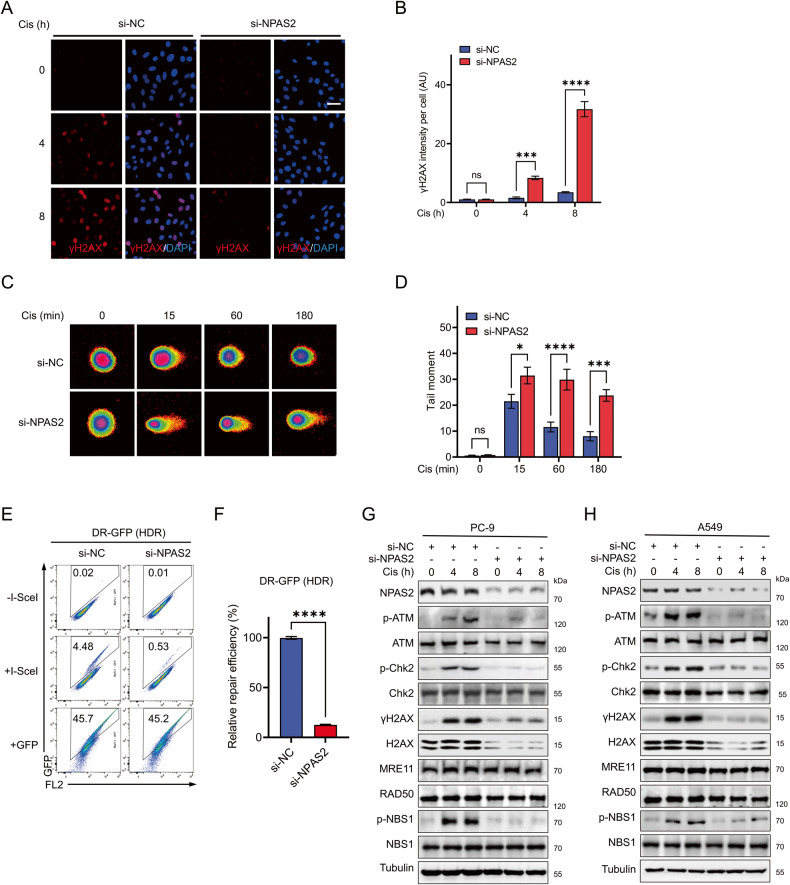
Depletion of NPAS2 impedes HDR by hindering the DNA damage repair signaling cascade. **A** Immunofluorescence micrographs of γH2AX in PC-9 cells transfected with si-NPAS2 and si-NC after the treatment of 10 µM cisplatin at the indicated time points, with nuclei counterstained by DAPI. Scale bar, 50 μm. **B** A quantitative analysis of the grayscale values of γH2AX foci per cell in (**A**). 10–20 random fields of view were counted at each time point. **C** Shown are representative micrographs of natural comet assay in PC-9 cells transfected with si-NPAS2 and si-NC after the treatment of 10 µM cisplatin for indicated time points. **D** Quantification of tail moments (TM values) of cells in (**C**). TM values of 20–40 single cells at each indicated time point were counted. **E** Flow cytometric detection of the effect of NPAS2 depletion on the efficiency of homology-directed repair. U2OS cells expressed with HDR reporter were transfected with I-SceI. The frequency of GFP-positive cells 48 h after transfection is shown, which represents the HDR efficiency. Cells transfected with GFP demonstrated the transfection efficiency, whereas cells that did not transfect with I-SceI were used as a blank control. **F** Quantification of **E** from three independent cell cultures. **G** and **H** PC-9 (**G**) and A549 (**H**) cells were transfected for 72 h with si-NC or si-NPAS2 before being treated with 10 µM cisplatin at the indicated time points. The protein levels of the indicated genes were determined using immunoblotting, with α-tubulin as a loading control. ns non-significant difference; *p* > 0.05; *p < 0.05; ***p < 0.001; ****p < 0.0001.

As described above, γH2AX plays a pivotal role in DNA damage repair, which serves as a docking site for DNA repair proteins. Upon repair of DSBs, γH2AX accumulates at the site of DNA damage to form foci. Immunocytochemistry results revealed that the deficiency of NPAS2 in PC-9 greatly impeded the accumulation of cisplatin-induced γH2AX (Fig. 3A, B), indicating an impaired DNA damage repair. Comet tail moments determined by the neutral comet assay could reflect the severity of DSBs in cells. The results showed that after cisplatin treatment, comet tails were reduced in a time-dependent manner in NPAS2-sufficient PC-9 cells. In contrast, comet tails failed to decrease during the same time period in NPAS2-deficient PC-9 cells (Fig. 3C, D), indicating impaired DSB repair.

The effectiveness of HDR was measured using the HDR reporter assay in U2OS cells that were stably transfected with HDR reporters. The HDR reporter contains recognition sites for the rare-cutting endonuclease I-SceI. Upon transfection with I-SceI in these cells, double-stranded DNA damage is induced. If homologous recombination is utilized for repair, the U2OS cells will express a green fluorescent protein (GFP), and the positivity of GFP, determined through flow cytometry analysis, serves as an indicator of the level of repair efficiency. Knockdown of NPAS2 significantly suppressed the repair efficiency of HDR, as measured by the HDR reporter assay (Fig. 3E, F). Notably, the transfection efficiency was unaffected after NPAS2 knockdown (Fig. 3E). Cisplatin initially causes interstrand crosslinks repaired via nucleotide excision repair (NER) and base excision repair (BER) mechanisms. The NER reporter assay was employed to assess the functionality of NER in U2OS cells. The findings unveiled that the downregulation of NPAS2 exerted no influence on the efficacy of NER. These findings strongly suggest that NPAS2 plays an indispensable role in the repair of double-stranded DNA breaks via HDR.

The results of immunoblotting showed that the phosphorylation of ATM, NBS1, Chk2, and H2AX was robustly induced by cisplatin and DSBs inducing agents, specifically doxorubicin and etoposide, in NPAS2-sufficient PC-9 cells (Fig. 3G, H, Supplementary Fig. 2A, B). In contrast, NPAS2-deficient cells showed a marked attenuation of the phosphorylation of these proteins, indicating that the DNA damage repair signaling pathway was compromised upon NPAS2 knockdown (Fig. 3G, H, Supplementary Fig. 2A, B). Furthermore, it was observed that the protein levels of ATM, NBS1, and Chk2 remained unchanged following knockdown of NPAS2. However, there was a decrease in the protein level of H2AX, which is consistent with the previously observed decrease in the mRNA level of H2AX.

### NPAS2 is essential for the stability of H2AX mRNA

**Fig. 4:**
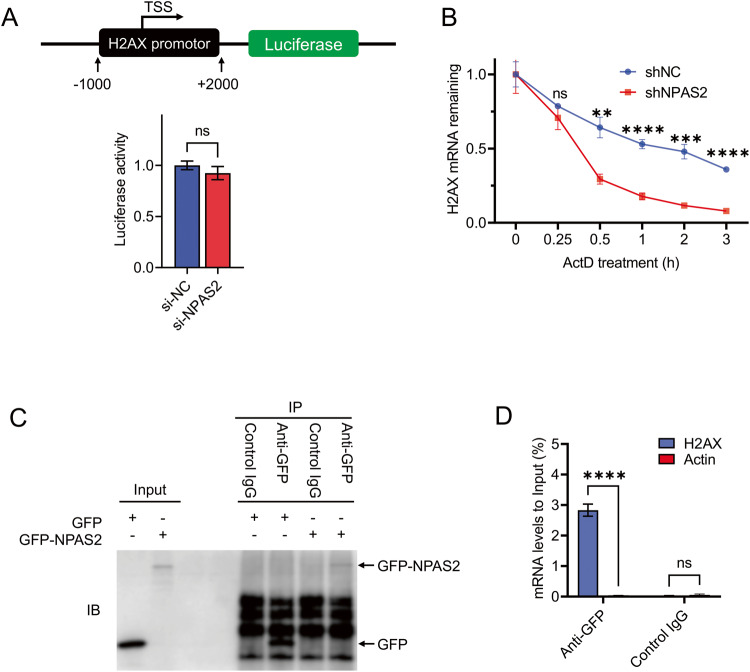
NPAS2 may increase the stability of H2AX mRNA by binding to its mRNA. **A** Luciferase activity of H2AX promoter constructs was measured in 293T cells. The ratio of reporter luciferase activity to control pRL-TK renilla luciferase activity is indicated. TSS transcription start site. **B** After actinomycin D treatment, the stability of H2AX mRNA in PC-9 cells was determined by RT-qPCR. **C** Cells that were transfected with GFP or GFP-NPAS2 were lysed and used for RIP assay based on GFP mouse antibody. Inputs and RNA immunoprecipitants were immunoblotted for GFP using GFP rabbit antibody (bottom). **D** RNA immunoprecipitation (RIP) assay shows that NPAS2 could bind with the mRNA of H2AX. ns non-significant difference; *p* > 0.05; **p < 0.01; ***p < 0.001; ****p < 0.0001.

The precise molecular mechanism through which NPAS2 regulates the expression of H2AX is currently unknown. To further explore this, we initially investigated whether NPAS2 could regulate the transcriptional activity of H2AX. We constructed a dual luciferase reporter gene system containing the H2AX transcription start site (−1000/+2200) and transfected it into both control and NPAS2 knockdown PC-9 cells to assess the transcriptional activity of H2AX. However, the results indicated that the knockdown of NPAS2 did not significantly decrease the transcriptional activity of H2AX (Fig. 4A).

Therefore, we wonder whether NPAS2 regulates the expression of H2AX at the post-transcriptional level. Actinomycin D was then used to block transcription in NPAS2 deficiency cells and control cells, and the mRNA level of H2AX was quantified by RT-qPCR at 1, 2, and 3 h after treatment. As expected, the results showed that the degradation of H2AX mRNA in the NPAS2 knockdown group was faster than that in the control group (Fig. 4B), indicating a decreased stability of H2AX mRNA in cells with NPAS2 depletion.

Furthermore, the RNA immunoprecipitation (RIP) assay based on GFP antibody was employed to determine whether NPAS2 could bind to H2AX mRNA. RIP followed by RT-qPCR showed that the GFP antibody was specifically enriched with mRNA of H2AX in the cells overexpressing the GFP-NPAS2 fusion protein (Fig. 4C, D). This suggests that NPAS2 may increase the stability of H2AX mRNA by binding to its mRNA.

### NPAS2 modulates the sensitivity of LUAD cells to DNA-damaging agents

**Fig. 5:**
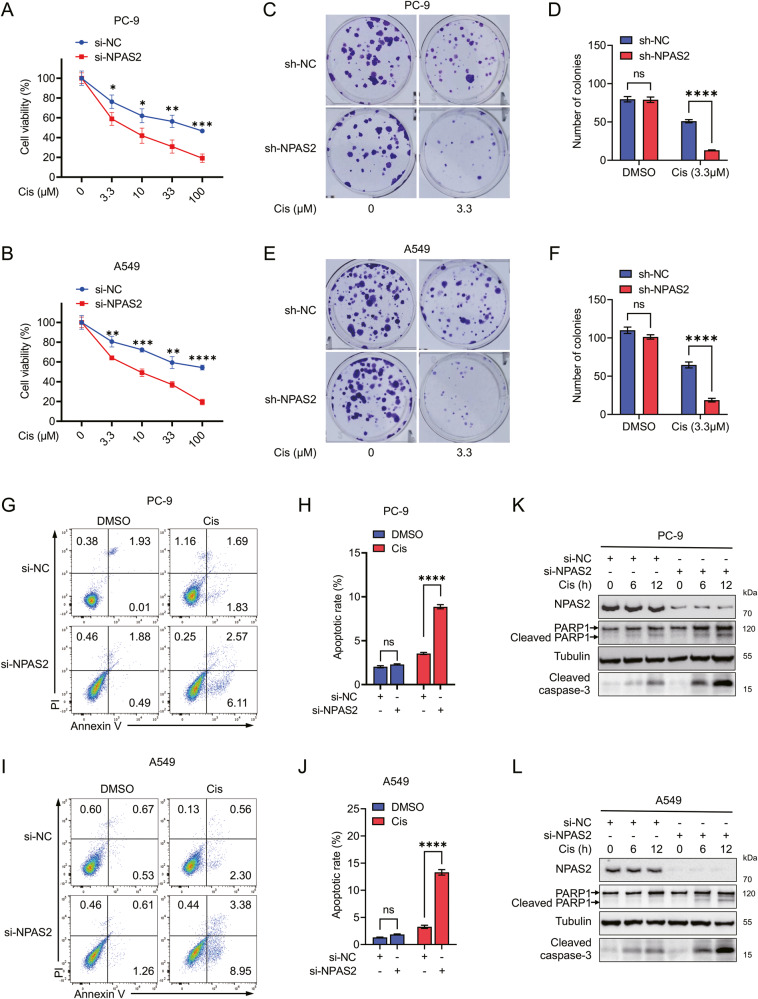
Knockdown of NPAS2 enhances the sensitivity of LUAD cells to cisplatin treatment. **A** and **B** PC-9 (**A**) and A549(**B**) cells were transfected with NPAS2-specific siRNA for 48 h, with the scramble siRNA as the internal control. The cell proliferation assay showed the survival fraction of each group after the treatment of the indicated dose of cisplatin for 72 h. **C**, **E** PC-9 and A549 cell lines infected with lentivirus carrying NPAS2-specific shRNA or scrambled non-specific shRNA control were employed for colony formation assays. These cell lines were treated with 3.3 μΜ cisplatin for 10 days before the cell colonies were counted. **D**, **F** The statistical analysis of the number of colonies in (**C**) and (**E**). **G**, **I** PC-9 and A549 transiently transfected with NPAS2 specific siRNA or scramble si-NC for 48 h and these cells were treated with 10 µM cisplatin for 24 h, then cells were subjected to fluorescence-activated cell sorting analysis after Annexin V-FITC/propidium iodide (PI) staining. Shown are representative flow cytometry analyses of apoptosis. **H**, **J** The statistical analysis of the percentage of apoptosis cells in (**G**) and (**I**). **K** and **L** The PC-9 (**K**) and A549 (**L**) cells were treated with 10 µM cisplatin for indicated times after the transfection with si-NC or si-NPAS2 for 72 h. The indicated proteins were measured by immunoblotting. Cis cisplatin, ns non-significant difference; *p < 0.05; **p < 0.01 ; ***p < 0.001; ****p < 0.0001.

Cell viability assays using MTT showed that LUAD cells with NPAS2 deficiency had significantly lower cell viability when exposed to equivalent amounts of cisplatin, doxorubicin, or etoposide compared to their control counterparts (Fig. 5A, B, Supplementary Fig. 2C, D). Whereas, amidst equivalent cisplatin-induced stress, the PC-9 and A549 with stable overexpression of GFP-NPAS2 showed significantly higher cell viability in contrast to their counterparts with GFP overexpression (Supplementary Fig. 3A, B). Additionally, the colony formation assay showed that the number of colonies formed in the NPAS2 knockdown group (sh-NPAS2) was significantly lower than that in the control group (sh-NC), when treated with the same dose of cisplatin, doxorubicin, or etoposide for a duration of 10 days (Fig. 5C–F and Supplementary Fig. 2E, F). Whereas, the cells with stable overexpression of GFP-NPAS2 exhibited a markedly increased colony formation compared to their GFP-overexpressing counterparts (Supplementary Fig. 3C–F). These results indicated a pivotal role of NPAS2 in the sensitivity of LUAD cells to DNA-damaging agents.

We observed a significant increase in cell apoptosis in the NPAS2 knockdown group compared to the control group when exposed to the same dose of cisplatin, as detected by Annexin V-FITC/PI via flow cytometry (Fig. 5G–J). However, the cells transiently transfected with Flag-NPAS2 displayed a noteworthy decrease in the rate of apoptosis compared to their Flag control counterparts (Supplementary Fig. 3G–J). Additionally, the deletion of NPAS2 resulted in a greater amount of cleaved-PARP1 and cleaved-caspase-3 in response to cisplatin treatment (Fig. 5K, L). These findings provide compelling evidence that NPAS2 is essential for the resistance of LUAD cells to cisplatin-induced apoptosis.

### NPAS2 deficiency xenografts are hypersensitive to DNA-damaging agents

**Fig. 6:**
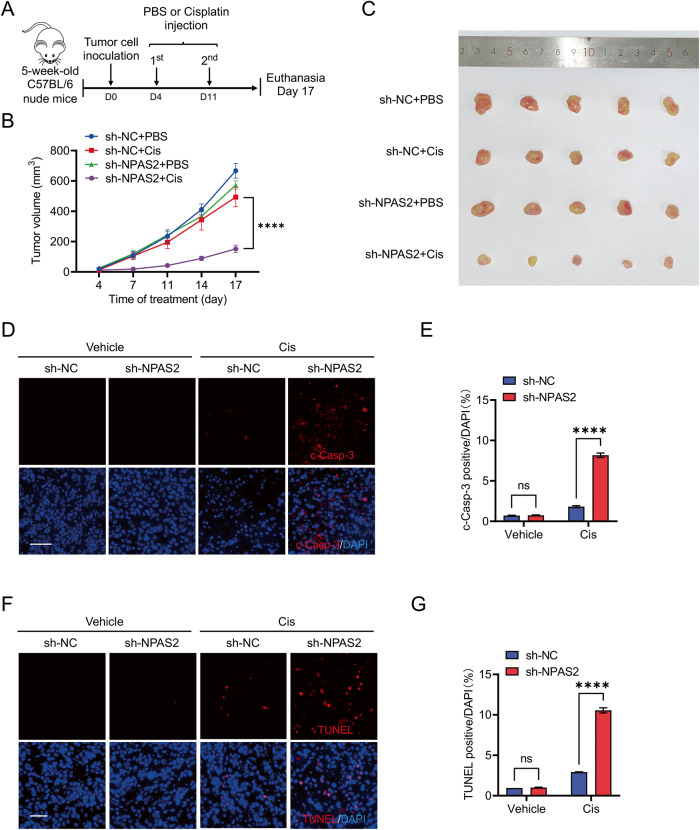
Xenografts with NPAS2 deficiency are more sensitive to DNA-damaging agents. **A** The experimental timeline for the subcutaneous xenograft assay is depicted schematically. **B** The tumor growth curve of the mice subcutaneously injected with PC-9 cells that transfected with sh-NC or sh-NPAS2 after intraperitoneal administration of cisplatin or PBS. **C** Typical images of tumors removed from mice on day 13 after implantation. **D** Immunofluorescence microscopy of cleaved-caspase-3 (red fluorescence) with DAPI-counterstained nuclei (blue fluorescence) in tumor sections of control or sh-NPAS2 PC-9 tumor xenograft. Scale bar, 100 μm. **E** Quantification of the percentage of cleaved-caspase-3 positive in the tumor sections. Data were summarized from five random fields. **F** The tumor sections’ TUNEL staining (red fluorescence) was used to assess the apoptosis in each group with DAPI-counterstained nuclei (blue fluorescence). Scale bar, 50 µm. Quantification of TUNEL staining in (**G**). Data were summarized from five random fields. ns non-significant difference; ****p < 0.0001.

In order to evaluate the impact of NPAS2 on cisplatin sensitivity, a subcutaneous xenograft assay was conducted using 5-week-old BALB/c nude mice, which were randomly injected subcutaneously with sh-NC and sh-NPAS2 PC-9 cells. When the xenografts became palpable 4 days after injection, the mice in each group were randomly divided into two subgroups, one receiving an intraperitoneal injection of a sub-effective dose of cisplatin (2.5 mg/kg) and the other receiving a vehicle control every 4 days, respectively (Fig. 6A).

The tumor growth curve of the mice in each subgroup was presented in Fig. 6B. In comparison to sh-NC PC-9 group, the sub-effective dose of cisplatin significantly inhibited the tumor xenograft growth in sh-NPAS2 group (Fig. 6C). The subcutaneous tumor tissue was stained with TUNEL (Fig. 6F, G) and cleaved-caspase-3 (Fig. 6D, E) was detected by immunohistochemistry. The results indicated that intraperitoneal injection of a sub-effective dose of cisplatin caused a greater amount of apoptosis in xenografts from the sh-NPAS2 group compared to those from the sh-NC group. These results suggested that NPAS2 can blunt the effect of cisplatin in LUAD.

## Discussion

The malignant condition of lung adenocarcinoma presents a formidable challenge, as it is associated with poor survival rates. Our preliminary bioinformatic analysis has revealed that the NPAS2 gene is highly expressed in LUAD and that patients in the high NPAS2 expression group exhibit poorer prognoses. These findings have been corroborated by the results from the GEO dataset and clinical samples, thus highlighting the significant role of NPAS2 in LUAD. In order to further investigate the possible mechanisms underlying NPAS2, mRNA sequencing was conducted to elucidate gene expression profiling following NPAS2 knockdown in PC-9 cells. Functional annotation analysis showed that the genes that were downregulated subsequent to NPAS2 knockdown were primarily enriched in DNA damage repair pathways.

Cellular responses to DNA damage are coordinated primarily by ATM–Chk2 and ATR–Chk1 axis, which includes the key factors such as ATM, ATR, Chk1, and Chk2, MRE11, NBS1, RAD50, etc. Further investigation revealed that NPAS2 knockdown resulted in a significant reduction in both mRNA and protein levels of H2AX while having no impact on the mRNA levels of ATM, ATR, Chk1, Chk2, MRE11, NBS1, and RAD50. We delved further into the specific molecular mechanisms by which NPAS2 affects DNA damage repair through regulating H2AX. Our dual-luciferase reporter assay indicated that NPAS2 did not affect the transcriptional activity of H2AX. However, the results of our mRNA stability assay using transcription inhibition by actinomycin D showed that depleting NPAS2 significantly increased the degradation of H2AX mRNA. Furthermore, our RIP combined with RT-qPCR assays revealed that NPAS2 could bind with the mRNA of H2AX. These findings indicate that NPAS2 can bind to and stabilize the mRNA of H2AX.

H2AX, a variant of the histone protein H2A, plays a critical role in the detection and repair of DSBs, the most harmful form of DNA damage. When DSBs occur, the HDR pathway is initiated, which involves the recognition and processing of the break by a complex of proteins, including the MRN complex and ATM. ATM then phosphorylates H2AX at serine 139 (γH2AX) in the vicinity of the breaks, which is a crucial early step in the HDR pathway. And in turn, this phosphorylation event is critical for the recruitment of repair factors, including the MRN complex, on the site of the DSBs. Without the phosphorylation of H2AX, the HDR pathway may not be efficiently activated and the repair of DNA DSBs could be impaired. Intriguingly, the GSEA revealed that downregulated genes following NPAS2 knockdown were enriched in the HDR pathway. By immunofluorescence assay, we found that γH2AX expression was significantly decreased in cisplatin-treated NPAS2 knockdown LUAD cells indicating a suppressed DNA damage repair. Meanwhile, the comet assay and HDR reporter assay also confirmed that the DNA damage repair process was dampened due to NPAS2 depletion. Further investigation revealed that phosphorylation of core proteins in response to DSBs such as ATM, Chk2, and NBS1 was severely attenuated in NPAS2-depleted cells.

Platinum-based chemotherapies, such as cisplatin, have remained a cornerstone of LUAD treatment and continue to play an essential role in clinical practice, despite remarkable progress in therapeutic strategies [22, 23]. The main mechanism of platinum-based chemotherapeutic agents is by interacting with bases on the DNA strand and thus forming interstrand crosslinks, which could be remedied via NER and/or BER mechanisms. Failure to aptly repair these crosslinks precipitates DNA double-strand breaks (DSBs), culminating in the inevitable cellular demise [24, 25]. While the rapid repair of DNA damage allows cells to escape cell death. Thus, the activation of the DNA damage repair pathway is one of the main reasons for the resistance of cancer cells to cisplatin [26, 27]. Within this study, the HDR reporter assay unveiled significant suppression in HDR repair efficacy following NPAS2 interference. However, the NER reporter assay indicated that NPAS2 downregulation wielded no discernible influence on the efficacy of NER, whilst a notable decline in NER efficiency was observed using a poly(ADP-ribose) polymerase (PARP) inhibitor, olaparib, which functioned as a positive control, given its ability to disrupt NER facilitation by PARP1 [28–30]. Considering that NPAS2 is involved in HDR, but not NER (Supplementary Fig. 1), we suspect that NPAS2 may mediate the cisplatin resistance in LUAD through regulating DSBs repair. The LUAD cells with knockdown of NPAS2 showed decreased survival and significantly increased apoptosis after cisplatin treatment, suggesting that the cells are hypersensitive to cisplatin. The results from the mouse model showed that the same dose of cisplatin administered to xenografts injected with sh-NPAS2 PC-9 cells resulted in significant inhibition of subcutaneous tumor growth, indicating increased sensitivity of tumor cells to chemotherapy drugs compared to sh-NC PC-9 cells.

Our findings suggest that NPAS2 plays a pivotal role in controlling of cisplatin sensitivity of LUAD cells by regulating the HDR pathway. The underlying mechanism involves the binding of NPAS2 to H2AX mRNA, which increases the stability of H2AX mRNA and subsequently promotes the DNA damage repair cascade, leading to the evasion of cell death in LUAD cells. The findings offer significant insights into the natural processes that contribute to cisplatin resistance in LUAD, providing an opportunity for researchers to better understand and overcome this challenge. The knowledge gained from these discoveries may pave the way for the development of innovative and effective strategies to combat cisplatin resistance and improve outcomes for LUAD patients.

## Materials and methods

### Data collection

The expression of the target gene in TCGA pancancer was explored using the online tools Gene Set Cancer Analysis (GSCA) [31]. Four external cohorts, including GSE36471 (*N* = 292) [32], GSE31210 (*N* = 158) [33], GSE42127 (*N* = 180) [34], and GSE72094 (*N* = 442) [35] were downloaded from the Gene Expression Omnibus (GEO) database.

### Real-time quantitative PCR (RT-qPCR)

The entire RNA content was extracted from both tumor tissues and cell lines, utilizing an RNA isolator (Vazyme, Nanjing, China). All-in-one 1st Strand cDNA Synthesis SuperMix Kit (Novoprotein, Jiangsu, China) was utilized to generate complementary DNA (cDNA) from RNA. The RT-qPCR was performed using Hieff® qPCR SYBR Green Master Mix (Yeasen, Shanghai, China) on the QuantStudio5 Real-Time PCR System (Applied Biosystems, USA). The relative expression levels of the target genes were normalized to endogenous ACTB. The target transcripts’ primer sequences are provided in Supplementary Table S1.

### Cell culture

PC-9, U2OS, 293T, and A549 were used in this study and these cell lines have been authenticated through STR profiling. Cell lines were maintained in DMEM (Servicebio, China) supplemented with 10% fetal bovine serum (Biological Industries, Beijing, China), and cultured in a humidified 5% CO_2_ incubator at 37 °C. Mycoplasma testing was conducted every 6 months using the GMyc-PCR Mycoplasma Test Kit (Yeasen, Shanghai, China).

### Plasmid vector construction and cell transfection

NPAS2-specific siRNAs were designed and synthesized by GenePharma company. The oligonucleotide sequences of NPAS2-specific siRNAs are provided in Supplementary Table S1. To construct the Flag-NPAS2 vector, the full-length NPAS2 coding sequence was amplified by PCR and subsequently ligated into the linearized pCMV-3×Flag vector. Transient transfection was performed using Lipofectamine 2000 reagent (Invitrogen; Thermo Fisher Scientific, Inc., USA) following the manufacturer’s instructions. For lentivirus production, lentiviral expression vectors and packaging plasmids (pMD2.G(Addgene No. 12259) and psPAX2(Addgene No. 12260)) were co-transfected into 293T cells. Following 48–72 h of transfection, the viral supernatant was collected, filtered, and used to infect PC9 and A549 cells. Infected cells were selected using puromycin and NPAS2 knockdown or overexpression was confirmed through Western blotting. shRNA expression vectors were created by cloning a specific sequence into LV3(pGLVH1/GFP+Puro) vector (GenePharma, Shanghai, China), which produces small hairpin RNA (5’- GAAAUUUGUAGGAAAUUUA-3’) for targeting the human NPAS2 mRNA sequence.

To construct the pPLJM1-EGFP-NPAS2 lentiviral vector, the NPAS2 coding sequence was amplified and ligated into the pPLJM1-EGFP vector (Addgene: Plasmid #19319). The primers employed for the construction of these vectors are provided in Supplementary Table S1.

### Expression profiling by high throughput sequencing

The PC-9 cells transfected with si-NPAS2 or scramble siRNA control were collected and frozen in liquid nitrogen before RNA extraction. Illumina sequencing libraries were generated and sequenced using the Illumina Novaseq 6000 system. The RNA-seq data were analyzed by first removing adapter sequences and low-quality reads from the raw reads to obtain clean reads. The clean reads were then mapped and aligned to the human genome (GRCH38) using HISAT2 [36]. The expression of genes was calculated using featureCounts [37]. To identify DEGs, the Deseq2 R package was utilized, with the cutoff value being |log_2_(Fold Change)| > 0.5 and adjust *p*-value < 0.01. KEGG pathway enrichment analysis and Gene Ontology (GO) analysis were performed based on the DEGs using the clusterProfiler R package.

### Immunoblotting

The cells were lysed using cytolysis buffer containing 1 M Tris–HCl (Ph 8.0), 1.5 mM NaCl, 0.5% sodium deoxycholate, 0.1 mM EDTA, and 1% NP-40. Subsequently, cell extracts were separated using SDS–PAGE. After separation, the proteins are transferred from the gel to a nitrocellulose filter membrane. Before being probed with the appropriate primary antibodies, non-fat dry milk or bovine serum albumin (BSA) was used to prevent nonspecific binding of the detection antibodies. The signal produced by the secondary antibody and enzyme-linked chemiluminescence substrate is captured using chemiluminescence. The primary antibodies utilized in this research and their corresponding working concentrations are listed in Supplementary Table S2.

### Neutral comet assays

The intensity of DSBs was determined using the Comet Assay Kit (Trevigen) following the manufacturer’s instructions. Briefly, 3 × 10^4^ cells suspended in cold PBS were added to 50 µl of 0.7% molten low-melt agarose at 37 °C. The cell mixtures were pipetted onto slides and incubated at 4 °C for 15 min to allow the agarose gels to solidify. Subsequently, the gels were treated with pre-chilled lysis solution for 1 h to lyse the cells and then unwound in an alkaline solution for another hour. Neutral electrophoresis was carried out in a horizontal chamber at 30 V for 20 min. DNA was stained using Propidium Iodide (PI) and the slides were photographed using a fluorescence microscope (Zeiss, Germany) after drying. The CometScore software was used to measure and analyze the length of the migrating portion of DNA (tail moment), which characterizes the degree of damage to the cellular DNA (Tri-Tek, USA) [38].

### DSBs repair reporter assay

The DSBs repair assays were carried out as previously described [39]. In brief, NPAS2-specific siRNAs were transfected into U2OS cells that expressed DR-GFP (DR-GFP/homologous-directed repair) for 24 h. In the DR-GFP expression vector, the I-SceI-GFP cassette divides the entire GFP into 5’ and 3’ GFP fragments. Cells were transfected again with I-SceI (DSBs production at unique I-SceI sites) or GFP (indicating transfection efficiency) plasmids for another 48 h. When I-SceI causes DBS homologous recombination repair, the downstream iGFP can be used as a template for repair and subsequent GFP expression [40]. Flow cytometry was used to determine the proportion of GFP-positive cells, which indicated the efficiency of Homologous-Directed Repair.

### NER assay

The NER assay was performed according to the previously described methodology [41]. Briefly, NER reporters were generated through the exposure of plasmids to UV-C light, inducing DNA damage that hinders the expression of fluorescent reporters by impeding transcription. The subsequent repair process mediated by NER restores the expression of the reporter genes.

### Measurement of apoptosis using flow cytometry

According to the manufacturer’s instructions, the Annexin V-FITC Apoptosis Detection Kit (Absin, Shanghai, China) was utilized to evaluate the level of cell apoptosis. Initially, the cells were harvested and washed with cold PBS. Then, they were resuspended in 300 μL of binding buffer at a concentration of 10^6^ cells/ml. The cell suspension was then subjected to a 10-min incubation on ice in the dark with Annexin V-FITC, followed by a 5-min incubation with PI. Finally, the samples were analyzed using a DxP Athena™ Cytometer (Cytek Biosciences, USA), as previously described [42].

### MTT assay and colony formation assays

3-(4,5)-dimethylthiahiazo (-z-y1)-3,5-diphenytetrazoliumromide (MTT) assay was used for the measurement of cell viability. Briefly, Cells were transfected with NPAS2-specific siRNA or a negative control scramble siRNA for 48 h. Then cells were collected and seeded in 96-well plates and treated with indicated drug concentration at 72 h post-transfection. 10% MTT (5 mg/ml) (Sigma) was added to each well, and incubated for an additional 4 h in the incubator. Triplex solution (0.012 M HCl, 10% SDS, and 5% isobutanol) was then added to each well. After the solvent of purple crystals, the solution was quantified at a wavelength of 570 nm, which indicated the cell viability [43].

PC-9 and A549 cells infected with either a lentivirus that induced expression of NPAS2-specific shRNA (sh-NPAS2) or a scrambled non-specific shRNA control(sh-NC) were employed for colony formation assays. Firstly, cells were seeded in six-well culture plates at a density of 6 × 10^2^ cells, and then cultured in a regular medium for 10 days. The tumor colonies were fixed using 4% paraformaldehyde and then stained with crystal violet.

### Dual-luciferase reporter gene

The genomic DNA from PC-9 cells was extracted following the instructions of the manufacturer (D1700-50, Solarbio, China). The primer sequences for amplifying the promoter region of H2AX are shown in Supplementary Table S1. The H2AX promoter regions were subcloned into the pTA-basic Firefly luciferase expression vector. 48 h after transfection of H2AX Firefly luciferase reporter plasmid, cells were lysed, and luciferase activity was measured using the Dual-Luciferase Reporter Gene Assay Kit (11402ES60, Yeasen, China). The Renilla Luciferase plasmid was used as an internal control. Each experiment was conducted three times.

### RNA immunoprecipitation

RIP was performed Following the instructions of the RIP kit (BersinBio). 293T cells were collected after the transfection with GFP or GFP-NPAS2 for 36 h and were lysed in a polysome lysis buffer that contained protease and RNase inhibitors. After removing the DNA, the lysed cells were incubated with an equivalent amount of anti-GFP Mouse antibody or control IgG at 4 °C for an overnight period, followed by an hour of incubation with protein A/G beads. The immunoprecipitated RNA was extracted using a mixture of phenol, chloroform, and isopentanol.

The isolated RNA then was analyzed using quantitative real-time PCR (qRT-PCR) to measure the relative amount of RNA present.

### Tumor xenograft mouse models

Five-week-old male BALB/c nude mice were purchased from Hunan SJA Laboratory Animal Co. Ltd. Prior to the inoculation of tumor cells, mice were randomly assigned to distinct groups using random numbers, with each group containing five mice. For cell-based xenograft tumor models, sh-NC or sh-NPAS2 PC-9 (2 × 10^6^) cells were resuspended in 100 µl PBS and were injected subcutaneously into the hind flank of these mice, which were housed in specific-pathogen-free settings at about 26 °C and 50% relative humidity. From day 4 onwards, mice were injected intraperitoneally with chemotherapeutic drugs or vehicle control once every 1 week. And the tumor size was measured using calipers every three days. The tumor volume was estimated using the equation *V* = *l* × *w*^2^ × 0.5 (*l*, length; *w*, width). 13 days after PC-9 cell injection, all animals were sacrificed, with their subcutaneous xenograft tumors removed and fixed in 4% paraformaldehyde. No blinding was done for the animal experiments. The sample size for the xenograft mouse experiment was not determined using any statistical method but was instead based on previous experience with similar studies.

### Immunocytochemistry and immunohistology

The immunocytochemistry was conducted in accordance with the previously described method [44, 45]. Initially, the cells were fixed with 4% paraformaldehyde and permeabilized with 0.5% Triton X-100 for 5 min. After blocking nonspecific binding, the appropriate primary antibodies were then added to the cells and incubated overnight at 4 °C. The cells were then stained with fluorescent dye-conjugated secondary antibodies for 2 h, followed by treatment with 4’,6-diamidino-2-phenylindole (DAPI, Absin) for 5 min at room temperature. Finally, the slides were cover-mounted for fluorescence microscopy using a microscope (Zeiss).

For immunohistochemistry, the xenograft tumor tissues were removed and fixed in 10% buffered formalin for 24 h. The tissues were then embedded in paraffin and cut into 5 mm sections. The tumor sections were dewaxed and rehydrated, followed by antigen retrieval. The sections were then incubated with appropriate primary antibodies at 4 °C overnight. Then the sections were incubated with fluorescent dye-conjugated secondary antibodies for 2 h at room temperature. Subsequently, the sections were stained with DAPI for 10 min and visualized using a fluorescence microscope (Zeiss).

### Terminal deoxynucleotidyl transferase dUTP nick end labeling (TUNEL) assays

The TUNEL assay was employed to assess cell apoptosis in the tumor tissues of the xenograft using the TUNEL BrightRed Apoptosis Detection Kit (A113-01, Vazyme), which is conducted in accordance with the previously described method [17]. Initially, the tumor sections were dewaxed and rehydrated, followed by a 30-min incubation with proteinase K at room temperature. Next, the TUNEL reaction mixture was added to the tumor sections and incubated in a humidified chamber for 60 min at 37 °C. Subsequently, the nuclei were counterstained with DAPI for 10 min. Finally, the section was examined under a fluorescence microscope (Zeiss).

### Statistical analysis

R software or GraphPad Prism (version 8.0) were both used for all statistical analysis. No statistical methods were used to predetermine the sample size. Paired *t*-tests, Student’s *t*-tests, or two-way repeated measures ANOVA were used to analyze group differences. Homogeneity of variance between groups was statistically compared. SEMs, or standard errors of means, were displayed in graphs. ns represents a nonsignificant difference, and *p*-value < 0.05 was used to determine whether a difference was significant.

### Supplementary information

Supplementary Information

Supplementary Fig.1

Supplementary Fig.2

Supplementary Fig.3

Supplementary Table S1

Supplementary Table S2

Original Data File

## Data Availability

The authors declare that all data supporting the findings of this study are available within the paper in the main text or the Supplementary file. Expression data from PC9 cells following NPAS2 knockdown can be accessed under PRJNA947505 in the sequencing read archive (SRA).
