# Supplementary material for: NPAS2 dampens chemo-sensitivity of lung adenocarcinoma cells by enhancing DNA damage repair
Source: Cell Death Dis. 2024 Jan 30;15(1):101. doi: 10.1038/s41419-023-06256-3 (PMC10827782; doi:10.1038/s41419-023-06256-3)
Supplement: Supplementary file 1 — Supplementary Information [file 41419_2023_6256_MOESM1_ESM.docx]

**SUPPLEMENTARY INFORMATION**

**Supplementary Figure 1 The downregulation of NPAS2 did not impact the efficiency of NER.** (A) U2OS cells, initially treated with 4µM olaparib or transfection with either si-NPAS2 or a control si-NC for a period of 24 hours, were subsequently transfected with a NER reporter plasmid. The frequency of GFP-positive cells 48 hours after transfection of NER reporter is shown, which represents the NER efficiency. Cells transfected with GFP demonstrated the transfection efficiency, whereas cells treated with olaparib as a positive control. (B) Quantification of (A) from three independent cell cultures. ns, non-significant difference; ****, p < 0.0001. NER, Nucleotide Excision Repair.

**Supplementary Figure 2 NPAS2 depletion impairs the DNA damage repair signaling cascade and renders LUAD cells more susceptible to DSBs inducing agents.** (A-B) PC-9 cells transfected with lentivirus carrying shNPAS2 or the scrambled control were treated with 10µM etoposide (A) and 1µM doxorubicin (B) at the specified time points. Immunoblotting was employed to evaluate the protein levels of the indicated genes, with α-tubulin as a loading control. (C-D) PC-9 infected with lentivirus carrying NPAS2-specific shRNA or scrambled non-specific shRNA control were treated with the indicated dose of etoposide and doxorubicin for 72h. The cell proliferation assay was performed using MTT assay. (E) PC-9 infected with lentivirus carrying NPAS2-specific shRNA or scrambled non-specific shRNA control were employed for colony formation assays. These cell lines were treated with 0.5µM etoposide and 40nM doxorubicin for 10 days before the cell colonies were counted. (F) The statistical analysis of the number of colonies in (E). ETO, etoposide; DOX, doxorubicin; ns, non-significant difference; ***, p < 0.001; ****, p < 0.0001.

**Supplementary Figure 3 Overexpression of NPAS2 reduces the sensitivity of LUAD cells to cisplatin treatment.** (A-B). The cell proliferation assay, utilizing PC-9 (A) and A549 (B) cells with stable overexpression of GFP-NPAS2 or GFP achieved through lentiviral vector-mediated stable overexpression, displayed the survival fraction of each group after the treatment with the indicated dose of cisplatin for 72 hours. (C, E) PC-9 and A549 cells, stable overexpression of GFP-NPAS2 or GFP, were treated with 10μΜ cisplatin for a duration of 10 days, following which the cell colonies were enumerated. (D, F) The statistical analysis of the number of colonies in (C) and (E). (G, I) PC-9 and A549 cells were transiently transfected with Flag-NPAS2 or Flag control for 48 hours. Subsequently, these cells were exposed to 40µM cisplatin for 24 hours, followed by fluorescence-activated cell sorting analysis after Annexin V-FITC/propidium iodide (PI) staining to assess apoptosis. Presented here are representative flow cytometry analyses exemplifying apoptosis. (H, J) The statistical analysis depicted the percentage of apoptotic cells in (G) and (I). Cis, cisplatin; ns, non-significant difference; ***, p < 0.001; ****, p < 0.0001.

**Supplementary Table S1** The oligonucleotides information.

**Supplementary Table S2** The antibodies and their working concentration used in this study.
