# Supplementary figures and images for: NPAS2 dampens chemo-sensitivity of lung adenocarcinoma cells by enhancing DNA damage repair
Source: Cell Death Dis. 2024 Jan 30;15(1):101. doi: 10.1038/s41419-023-06256-3 (PMC10827782; doi:10.1038/s41419-023-06256-3)

Fig.3G

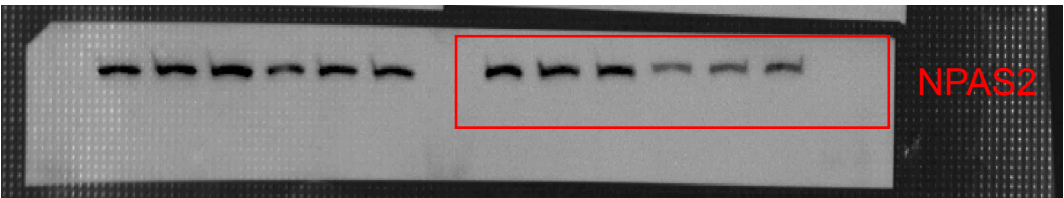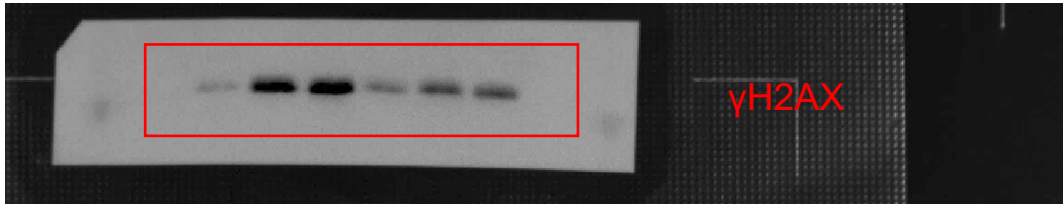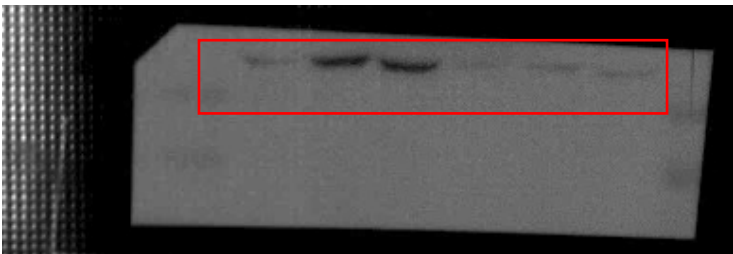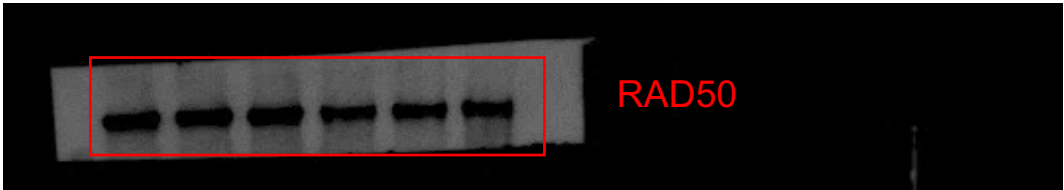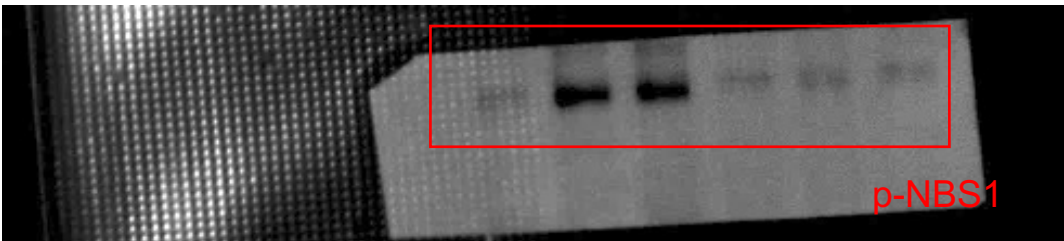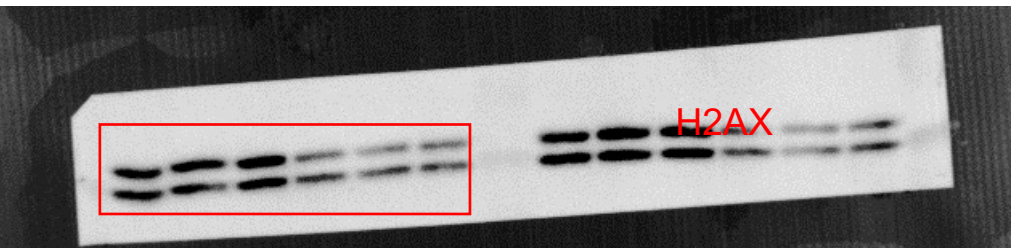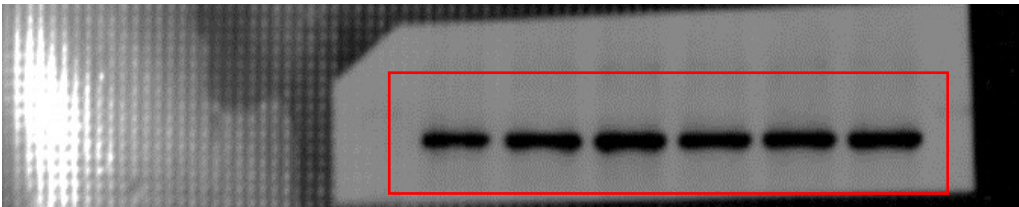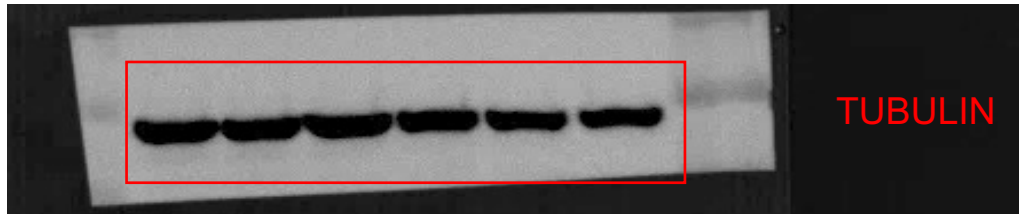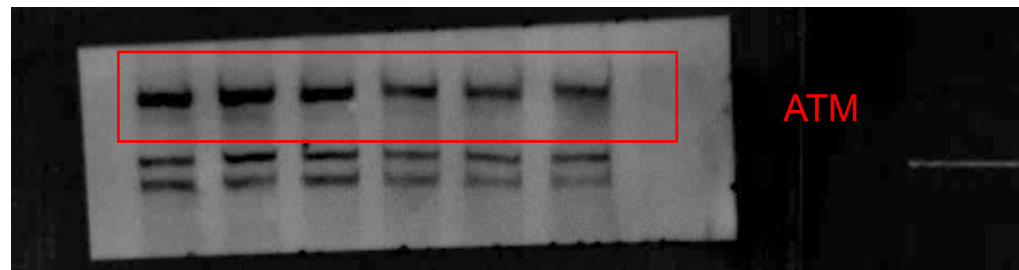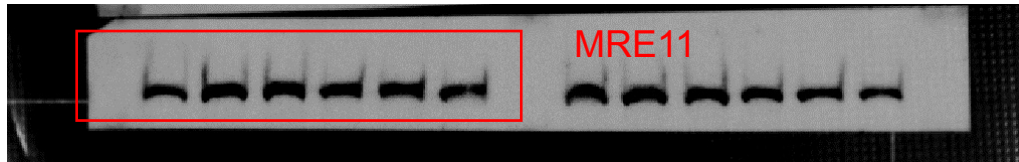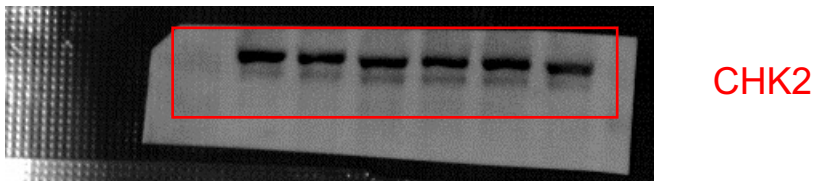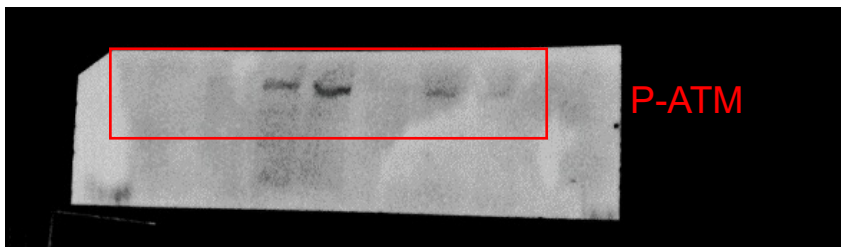

Fig.3H

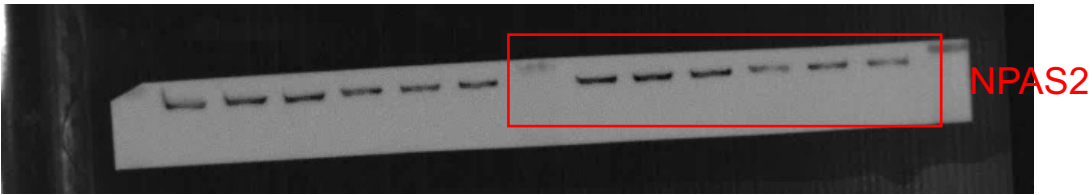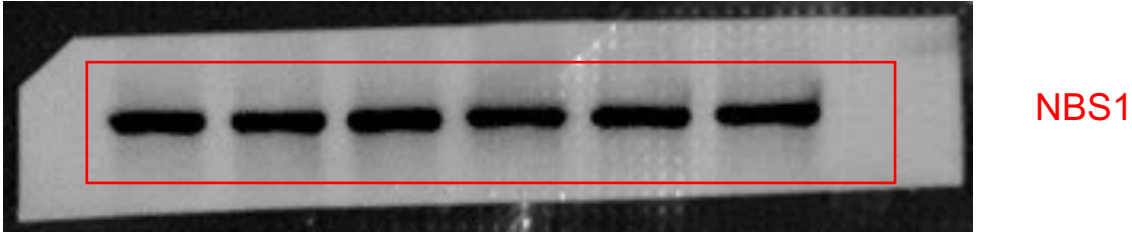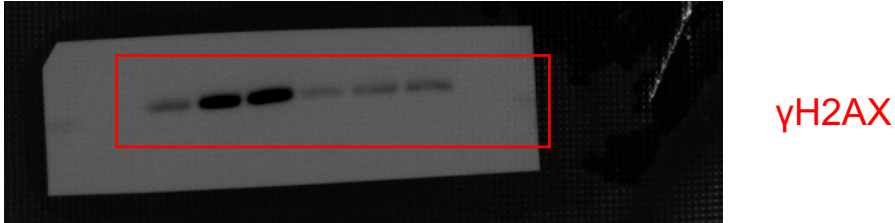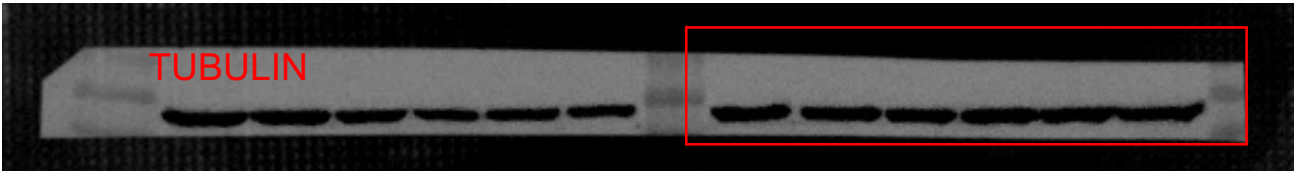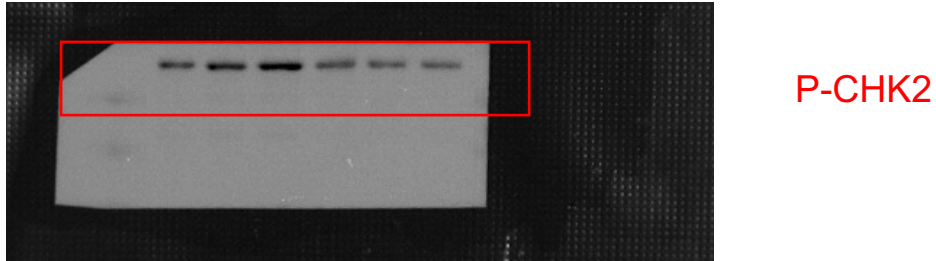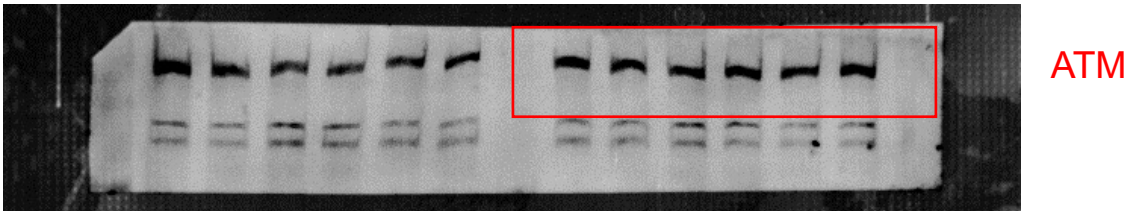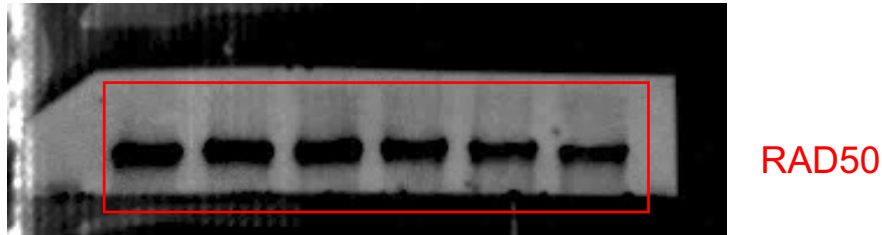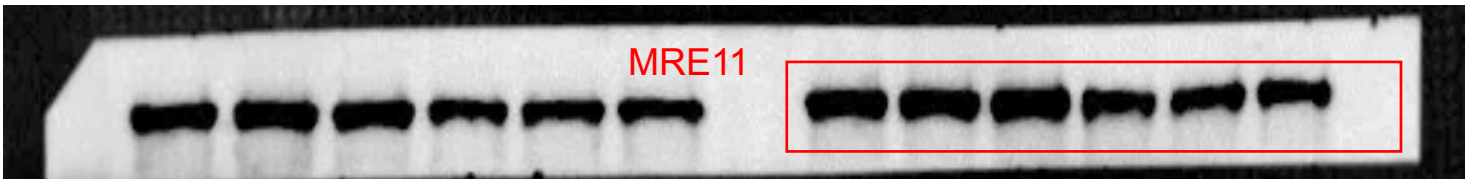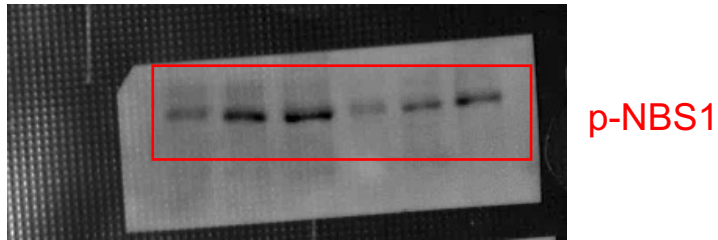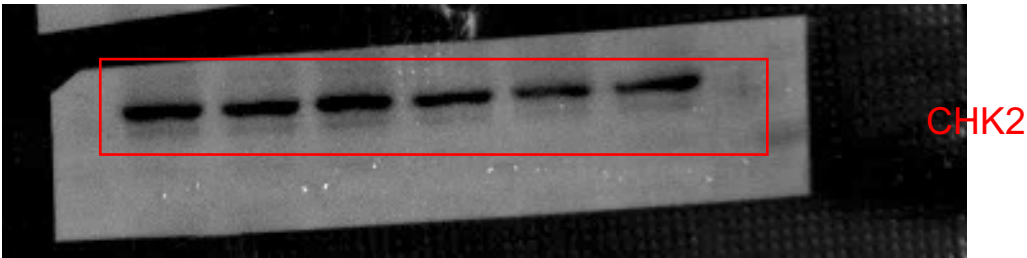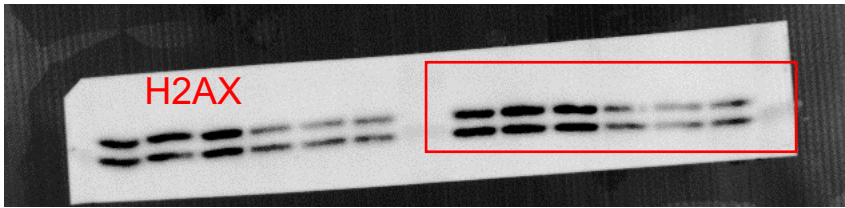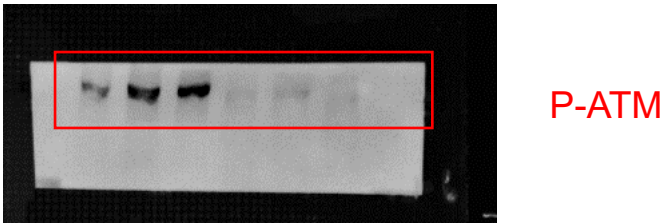

Fig.4C

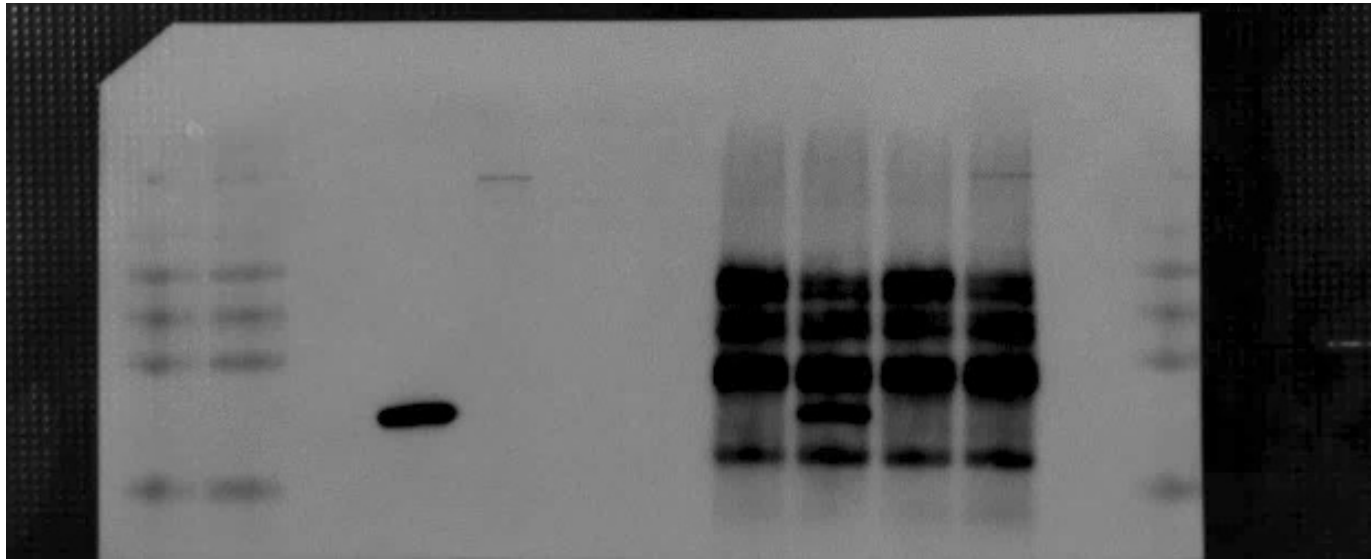

Fig.5K

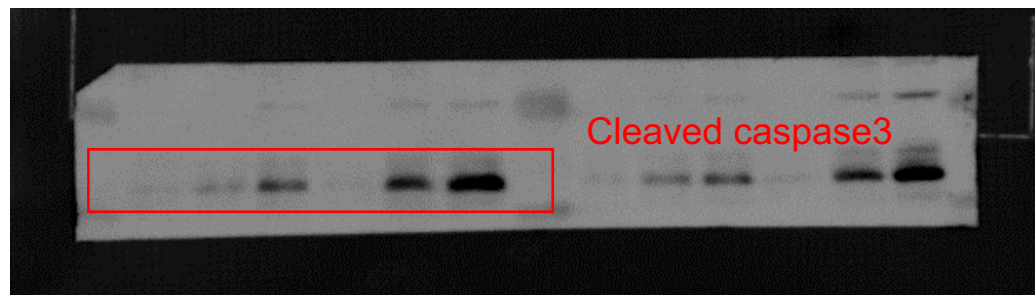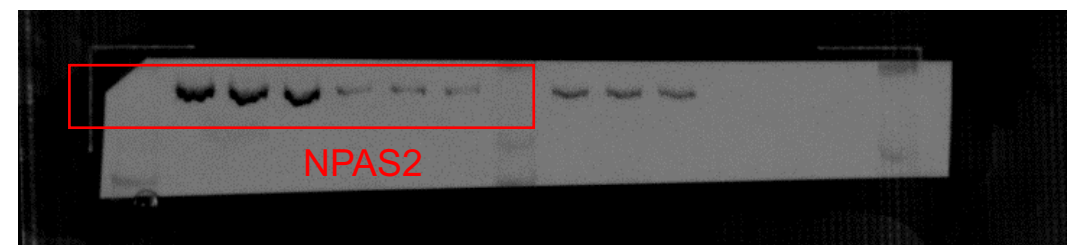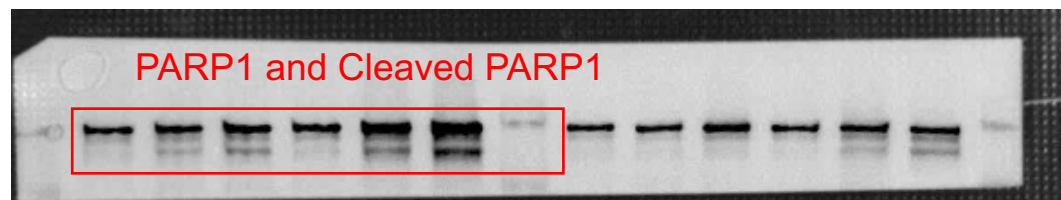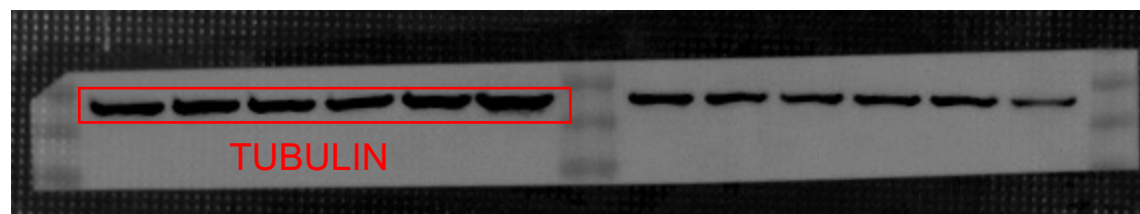

Fig.5L

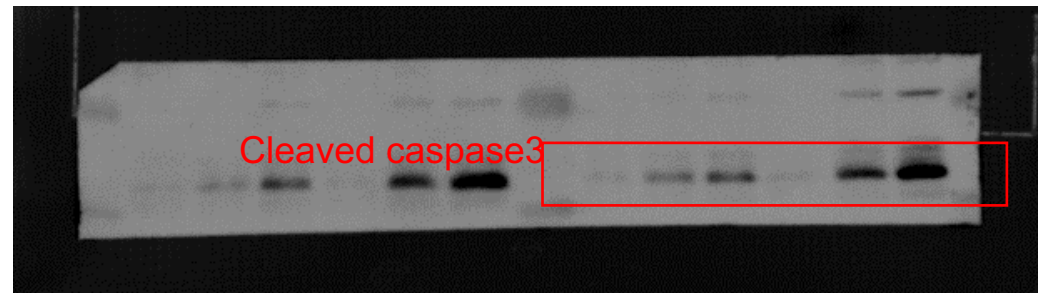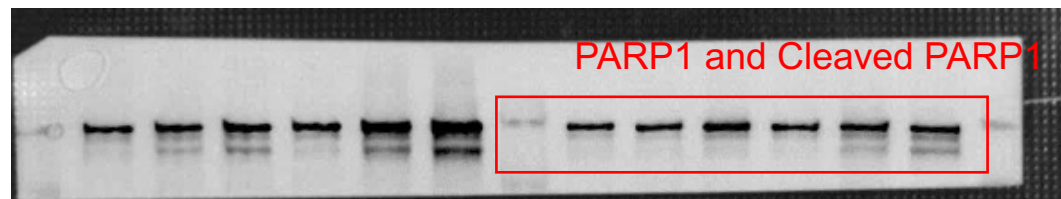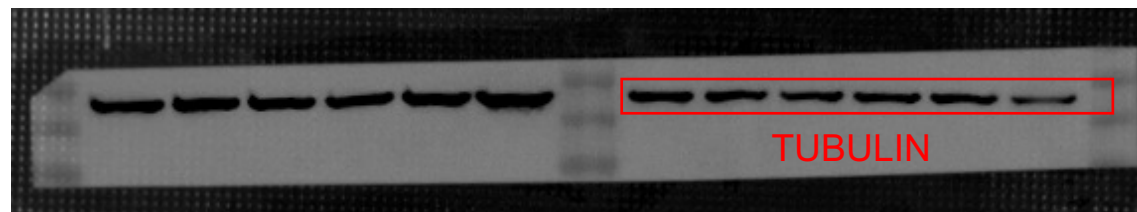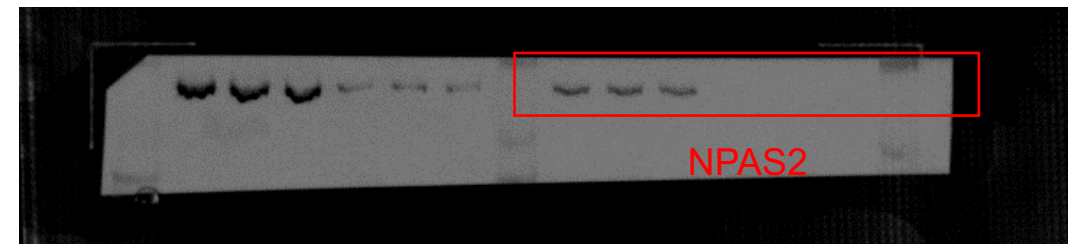

# Supplementary Figure 2A

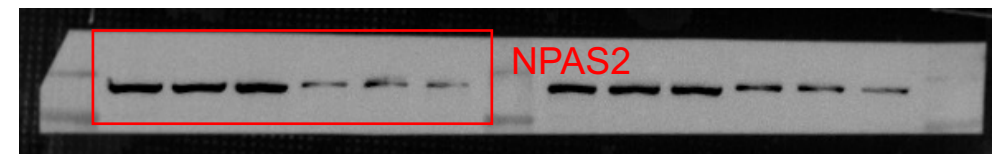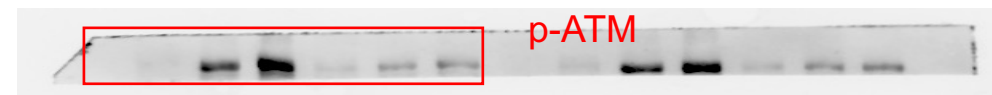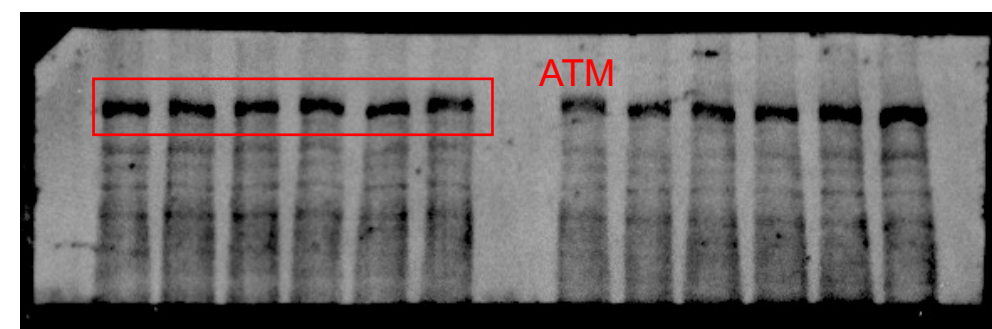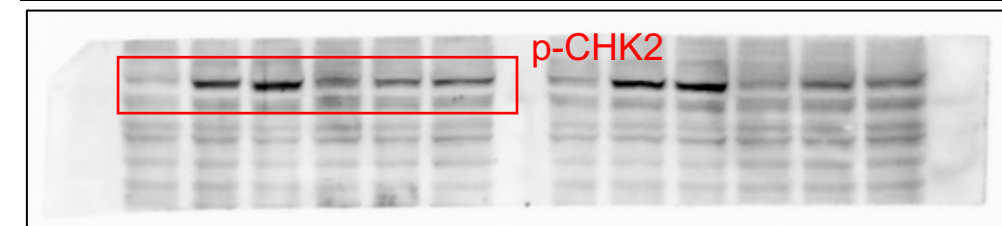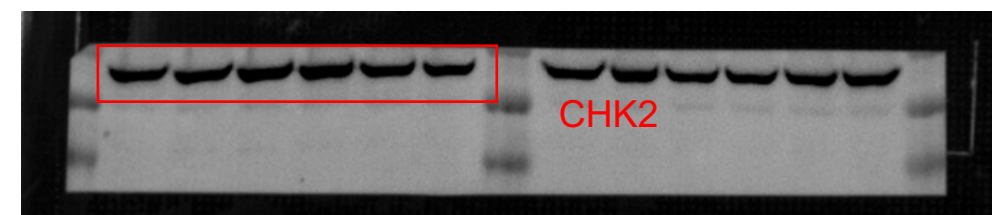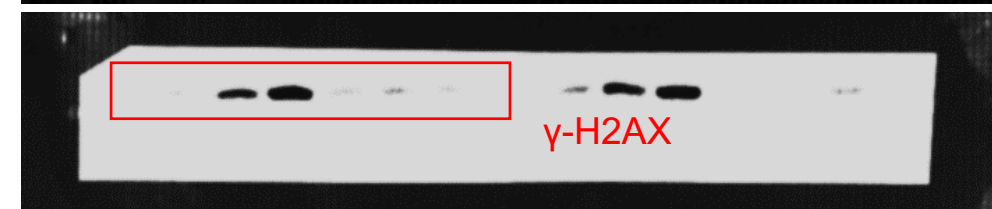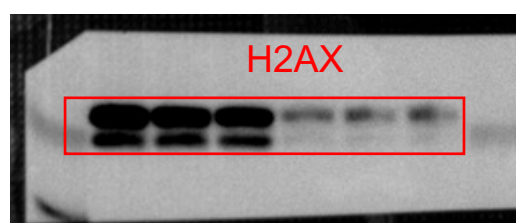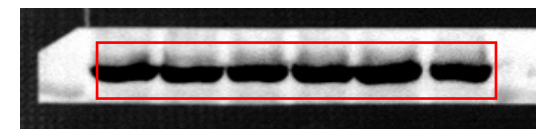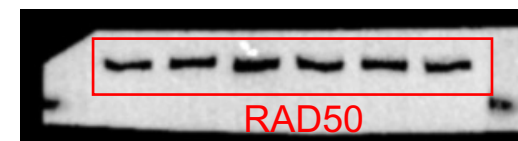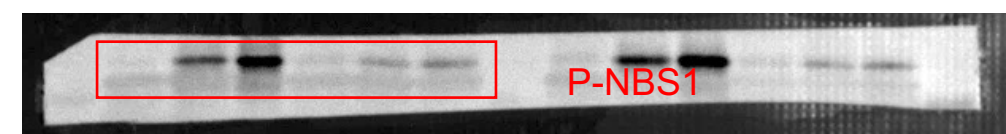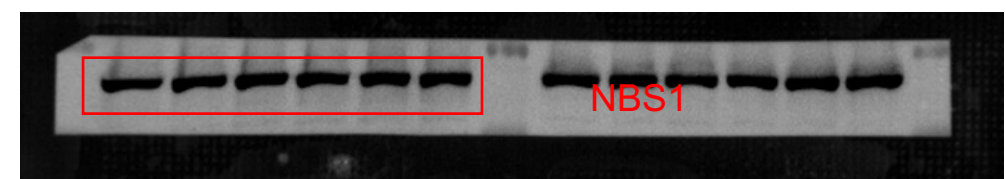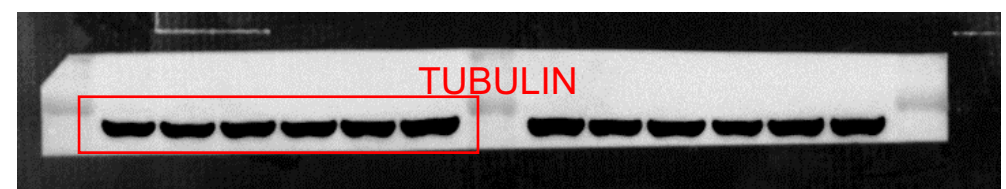

# Supplementary Figure 2B

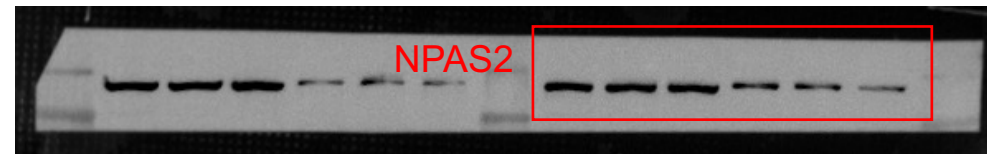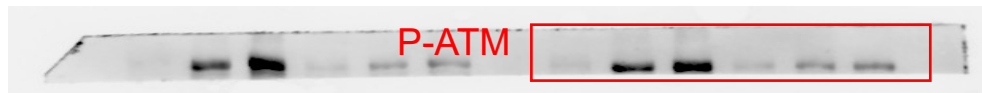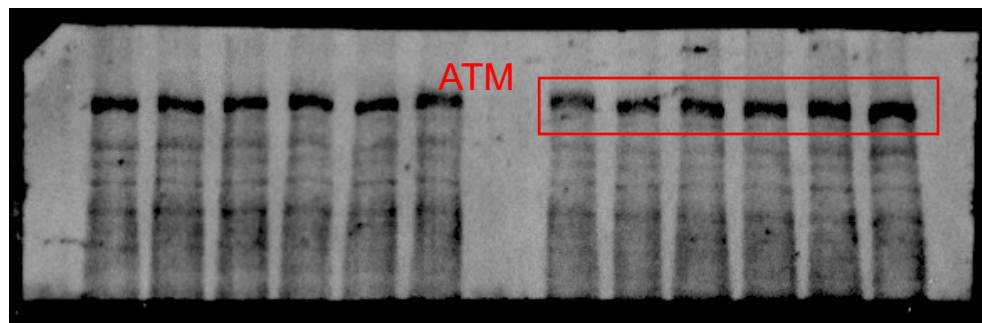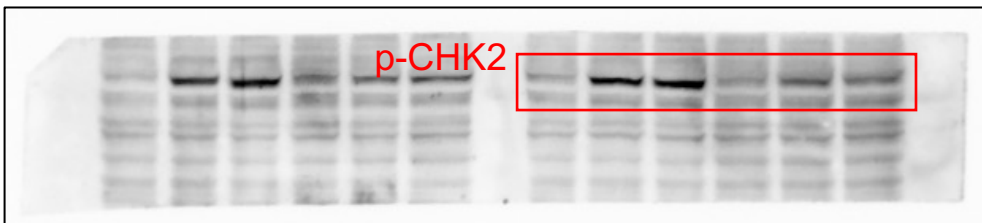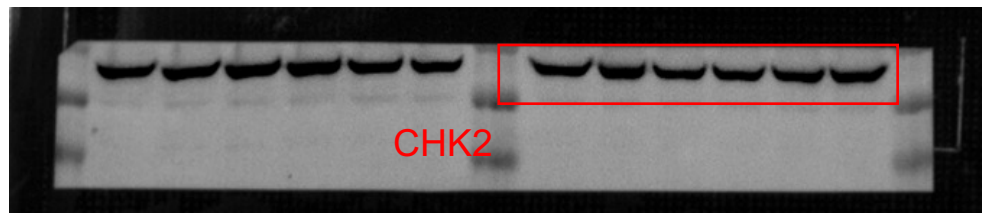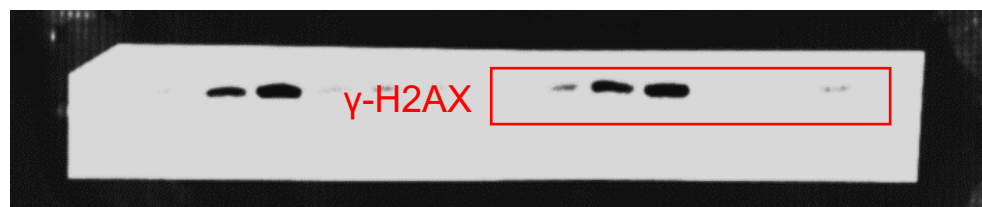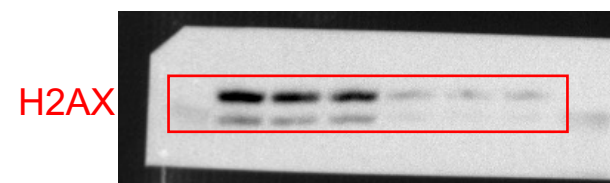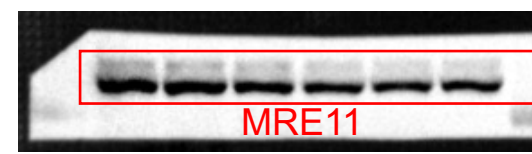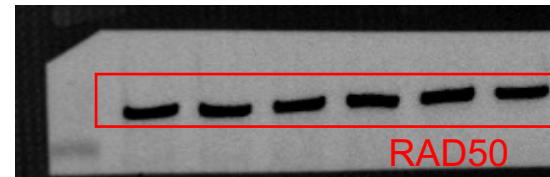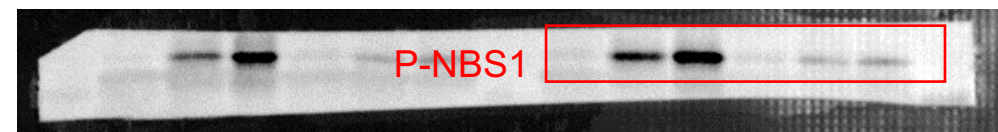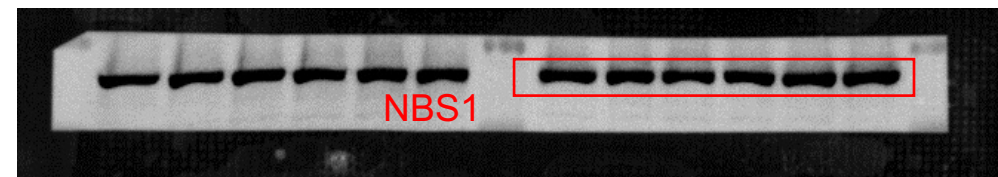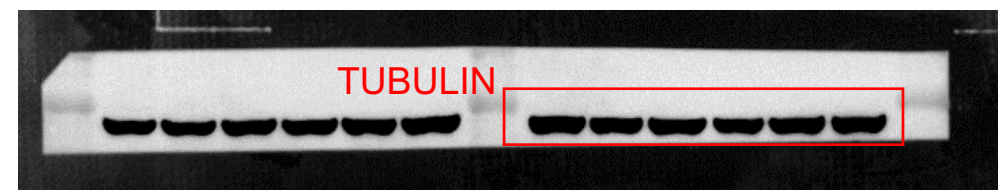

Supplement: Supplementary file 7 — Original Data File [file 41419_2023_6256_MOESM7_ESM.pdf]
